# Clinical Outcomes and Exploratory Longitudinal CTL/Vβ Repertoire Remodeling in Patients with Relapsed or Refractory Large B-Cell Lymphoma and Follicular Lymphoma Treated with Epcoritamab

**DOI:** 10.3390/ijms27115132

**Published:** 2026-06-05

**Authors:** Tatsuro Jo, Jun Taguchi, Yasushi Sawayama, Masatoshi Matsuo, Kaho Umemoto, Kaori Yamaguchi, Kazuhiro Noguchi, Takahiro Sakai, Saori Ikegami, Rena Baba, Tomoya Inoue, Sadaharu Irie, Kuniko Abe, Kazuto Shigematsu, Yasushi Miyazaki

**Affiliations:** 1 Department of Hematology, Japanese Red Cross Nagasaki Genbaku Hospital, Mori-machi 3-15, Nagasaki City 852-8511, Nagasaki, Japan; 2 Department of Clinical Laboratory, Japanese Red Cross Nagasaki Genbaku Hospital, Mori-machi 3-15, Nagasaki City 852-8511, Nagasaki, Japan; 3 Department of Pharmacy, Japanese Red Cross Nagasaki Genbaku Hospital, Mori-machi 3-15, Nagasaki City 852-8511, Nagasaki, Japan; 4 Department of Pathology, Japanese Red Cross Nagasaki Genbaku Hospital, Mori-machi 3-15, Nagasaki City 852-8511, Nagasaki, Japan

**Keywords:** large B-cell lymphoma, follicular lymphoma, epcoritamab, cytotoxic T lymphocyte, T-cell receptor repertoire, flow cytometry, CNS involvement

## Abstract

Epcoritamab, a subcutaneous CD3×CD20 bispecific antibody, has shown substantial activity in relapsed or refractory (R/R) B-cell lymphomas, but the immunological correlates of durable remission and treatment discontinuation remain unclear. We retrospectively analyzed 21 consecutive patients who initiated epcoritamab at our institution between 1 December 2023 and 31 December 2025, including 17 with R/R large B-cell lymphoma (LBCL) and 4 with R/R follicular lymphoma (FL). Clinical follow-up was updated through 18 May 2026. Serial cytotoxic T lymphocyte (CTL) subset and T-cell receptor (TCR) Vβ repertoire analyses were performed in selected cases. Among response-evaluable patients, the overall response rate was 9/14 in LBCL and 4/4 in FL. Median overall survival was 431 days in LBCL and 431.5 days in FL. Progression-free survival was analyzed descriptively because of the small sample size and substantial censoring. A patient with clinically and radiologically suspected central nervous system relapse of LBCL achieved radiological complete remission after epcoritamab treatment. In two LBCL and one FL case in whom epcoritamab was electively discontinued after complete remission, Vβ-skewed CTL populations were observed, and total memory CTLs exceeded total effector CTLs at discontinuation. These exploratory findings suggest that epcoritamab treatment may be associated with longitudinal remodeling of CTL subsets and Vβ-skewed CTL populations in selected responders. The potential relevance of these immunological patterns to durable response and treatment discontinuation should be validated in larger prospective cohorts with functional and sequence-based T-cell analyses.

## 1. Introduction

Treatment of relapsed or refractory (R/R) large B-cell lymphoma (LBCL) and follicular lymphoma (FL) remains a substantial clinical challenge despite recent advances in immunotherapy. Although anti-CD20-based chemoimmunotherapy, chimeric antigen receptor T-cell (CAR-T) therapy, and more recently bispecific antibodies have expanded the therapeutic landscape, a considerable proportion of patients with multiply relapsed disease still experience treatment resistance, early progression, or limited durability of response. Therefore, the identification of therapeutic strategies capable not only of inducing remission but also of establishing sustained immunologic control remains an unmet need in R/R B-cell lymphomas.

Epcoritamab is a subcutaneously administered CD3×CD20 bispecific antibody that redirects endogenous T-cells toward CD20-positive malignant B-cells. In the pivotal phase I/II study in patients with R/R LBCL, including diffuse large B-cell lymphoma (DLBCL), high-grade B-cell lymphoma (HGBCL), and primary mediastinal B-cell lymphoma (PMBCL), who had received at least two prior lines of therapy including anti-CD20 antibody-based treatment, epcoritamab achieved an overall response rate (ORR) of 63.1% and a complete remission (CR) rate of 40.1% [1]. With extended follow-up, the 2-year progression-free survival (PFS) and overall survival (OS) rates were 27.8% and 44.6%, respectively [2], whereas the median OS at 3 years was 18.5 months [3]. Importantly, among patients who achieved CR, remission durability was remarkable, with 2- and 3-year CR maintenance rates of 64.2% and 53%, respectively [2,3]. These findings suggest that, although only a subset of patients attains deep remission, those who do may derive prolonged clinical benefit.

Similarly, epcoritamab has demonstrated marked activity in R/R FL. In the EPCORE NHL-1 phase II cohort of patients with FL previously treated with at least two lines of therapy, epcoritamab yielded an ORR of 82.0% and a complete metabolic response rate of 62.5% [4]. Notably, favorable activity was also observed in patients with progression of disease within 24 months (POD24), a clinically high-risk subgroup generally associated with inferior outcomes [4]. Nevertheless, these encouraging response rates should not obscure the important clinical reality that patients who fail to achieve complete remission remain at increased risk of primary refractoriness, suboptimal disease control, or early relapse. Thus, elucidation of the immunological features associated with deep and durable response is of considerable clinical and biological importance.

From the standpoint of tolerability, epcoritamab has shown a manageable safety profile. Cytokine release syndrome (CRS) occurred in 49.7% of patients with R/R LBCL and 66.4% of those with R/R FL, but the great majority of events were grade 1–2, whereas grade 3 CRS was uncommon, occurring in only 2.5% and 1.6% of patients, respectively [1,4]. Immune effector cell-associated neurotoxicity syndrome (ICANS) was also infrequent, being reported in 6.4% of patients with R/R LBCL and 6.3% of those with R/R FL; grade 3 events were rare or absent [1,4]. Infections and neutropenia remain clinically relevant toxicities, but these events have generally been manageable with appropriate supportive care [1,4]. Moreover, a matching-adjusted indirect comparison suggested that epcoritamab may be associated with significantly lower rates of grade ≥3 CRS and ICANS than axicabtagene ciloleucel, supporting its potential as an effective and more broadly deliverable immunotherapeutic platform [5].

Mechanistically, the antitumor activity of epcoritamab is predicated on the physical juxtaposition of T-cells and CD20-positive lymphoma cells, thereby promoting immune synapse formation and subsequent T-cell activation, proliferation, and cytotoxicity. Preclinical studies have shown that CD3×CD20 bispecific antibodies can induce potent T-cell-mediated killing of malignant B-cells even under conditions of low baseline T-cell abundance [6]. Clinical correlative analyses have further shown that epcoritamab administration is associated with rapid expansion of peripheral CD4^+^ and CD8^+^ T-cells, a reduction in naïve T-cell fractions, and an increase in effector memory T-cell populations [1]. These observations raise the possibility that epcoritamab does not merely redirect pre-existing T-cells, but may also dynamically reshape the systemic T-cell compartment toward a more activated and functionally differentiated state.

By contrast, resistance to CD3×CD20 bispecific antibody therapy appears to be multifactorial and may involve both tumor-intrinsic and immune-mediated mechanisms. Proposed mechanisms include reduced or lost CD20 expression, the emergence of resistant subclones, impaired T-cell fitness, persistence of an immunosuppressive tumor microenvironment, and failure to achieve deep molecular or minimal residual disease-negative responses [1,4,7,8,9,10,11]. Collectively, these findings suggest that the quality of the antitumor T-cell response, rather than numerical T-cell expansion alone, may be a critical determinant of treatment efficacy. Beyond epcoritamab, other CD3×CD20 bispecific antibodies, including glofitamab, mosunetuzumab, and odronextamab, have also demonstrated clinically meaningful activity in R/R B-cell lymphomas. These agents collectively support CD3×CD20 bispecific antibodies as an important off-the-shelf T-cell-redirecting therapeutic class across aggressive and indolent B-cell lymphomas [12,13,14,15].

We have previously explored longitudinal CTL subset and TCR Vβ repertoire patterns in several hematologic malignancies, including B-cell acute lymphoblastic leukemia (B-ALL), adult T-cell leukemia/lymphoma (ATLL), and chronic-phase chronic myeloid leukemia (CML) [16,17,18,19,20]. Although these diseases differ substantially from B-cell lymphomas in biology and treatment context, these studies provided a rationale for exploring whether longitudinal CTL/Vβ repertoire changes may also accompany clinical responses to T-cell-redirecting therapy. Therefore, in the present study, we evaluated clinical outcomes and exploratory immune monitoring findings in patients with R/R LBCL and FL treated with epcoritamab.

## 2. Results

### 2.1. Patient Characteristics

The clinicopathological characteristics of the study cohort are summarized in Table 1. Between 1 December 2023 and 31 December 2025, 21 patients received epcoritamab at our institution, including 17 with R/R LBCL and 4 with R/R FL. Patients with R/R LBCL were aged 53–93 years (median, 80 years), whereas those with R/R FL were aged 63–79 years (median, 71 years).

In the LBCL cohort, the histological subtypes included DLBCL of germinal center B-cell type (*n* = 2), DLBCL of activated B-cell type (*n* = 1), DLBCL, not otherwise specified (*n* = 3), high-grade B-cell lymphoma (*n* = 6), T-cell-rich LBCL (*n* = 1), FL grade 3B (*n* = 1), and DLBCL transformed from FL (*n* = 3). All FL cases were grade 1–2.

Regarding prior lines of therapy, 12 patients with R/R LBCL had received ≤3 prior lines and 5 had received ≥4 lines, whereas in the R/R FL cohort, 2 patients had received ≤3 lines and 2 had received ≥4 lines.

Overall, the cohort represented a clinically high-risk real-world population, particularly in the LBCL group, which included elderly patients, frequent poor performance status, advanced-stage disease, extranodal involvement, and high IPI scores. All patients had confirmed CD20 expression, and none had received prior CAR-T-cell therapy. In patients with DLBCL transformed from FL, prior treatment lines included therapies administered for both antecedent FL and transformed DLBCL.

Treatment exposure and response outcomes are summarized in Table 2. At the data cutoff of 18 May 2026, 8 patients with R/R LBCL and 1 with R/R FL remained on epcoritamab treatment. Among 14 response-evaluable LBCL patients, 5 achieved complete response (CR) and 4 achieved partial response (PR), corresponding to an overall response rate of 9/14 (64.3%). Among the 4 patients with FL, all 4 responded, including 2 CRs and 2 PRs. Among 17 patients with LBCL, 3 were not evaluable for response because of early discontinuation before the first scheduled response assessment or insufficient imaging follow-up.

### 2.2. Reasons for Epcoritamab Discontinuation

The reasons for epcoritamab discontinuation are summarized in Table 3. Among patients with R/R LBCL, 2 discontinued treatment after CR had been confirmed following 21 cycles of epcoritamab. At the data cutoff of 18 May 2026, these patients had maintained CR for 9 and 6 months, respectively, after treatment cessation. One additional patient discontinued treatment because of sudden death of unknown cause despite being in CR. Another patient discontinued epcoritamab because of herpes zoster while in PR. Treatment was also discontinued in 3 patients because of death due to progressive disease (PD) and in 1 patient because of death from aspiration pneumonia. In addition, 1 patient discontinued epcoritamab after the first cycle at the patient’s request. In the R/R FL cohort, 1 patient discontinued epcoritamab after confirmation of CR following 12 cycles and remained in CR for 1 month at the data cutoff. The remaining 2 patients discontinued treatment because of COVID-19 infection, including 1 patient in CR and 1 in PR at the time of discontinuation.

### 2.3. Survival After Epcoritamab Treatment

**Figure 1:**
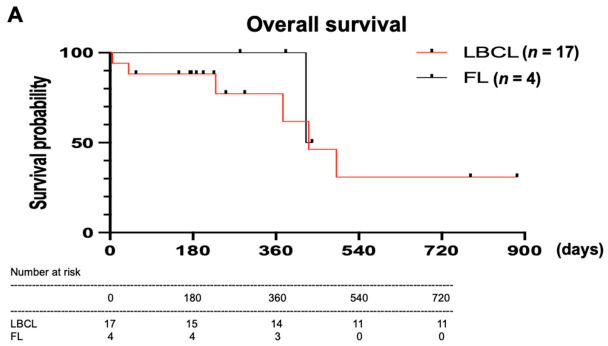

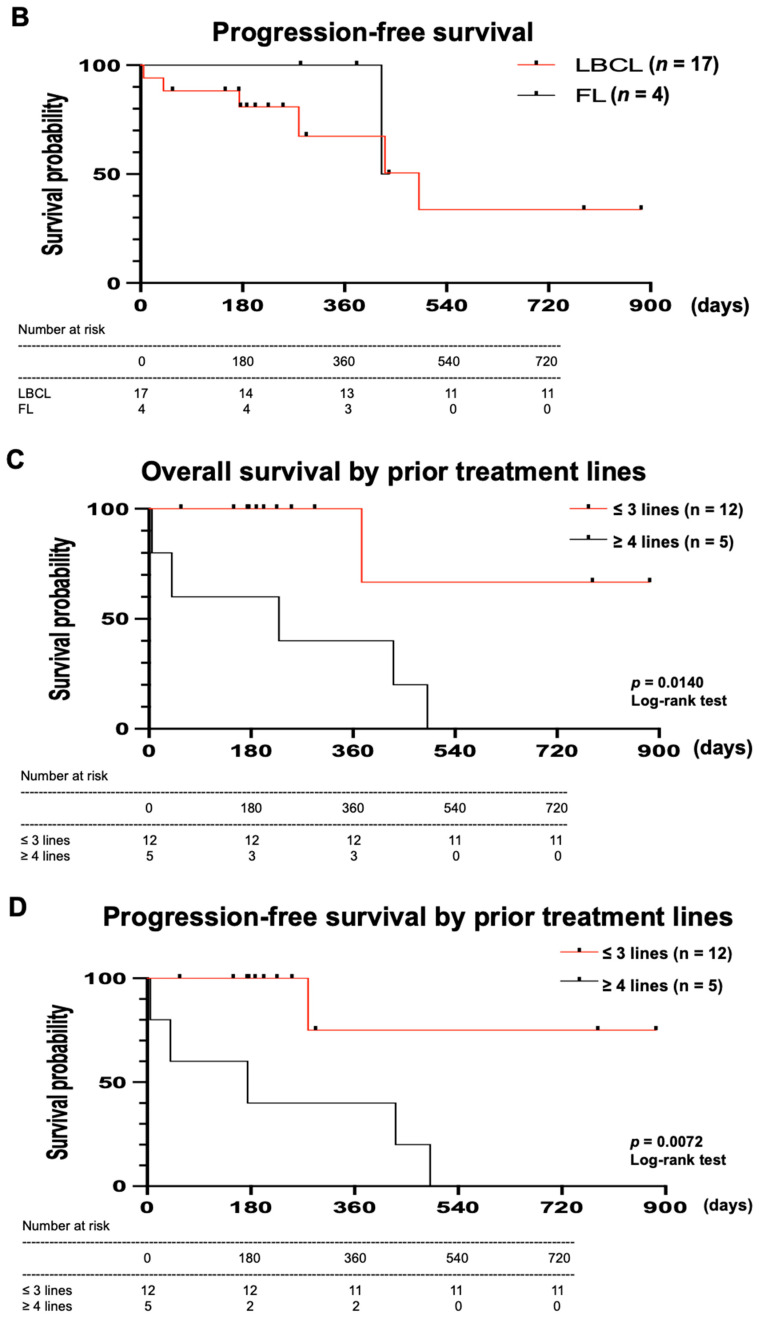
Overall survival and progression-free survival of patients treated with epcoritamab. (**A**) Kaplan–Meier curves of overall survival in patients with LBCL (*n* = 17) and FL (*n* = 4). (**B**) Kaplan–Meier curves of progression-free survival in patients with LBCL (*n* = 17) and FL (*n* = 4). (**C**) Kaplan–Meier curves of overall survival in patients with LBCL stratified according to the number of prior treatment lines: ≤3 lines (*n* = 12) and ≥4 lines (*n* = 5). (**D**) Kaplan–Meier curves of progression-free survival in patients with LBCL stratified according to the number of prior treatment lines: ≤3 lines (*n* = 12) and ≥4 lines (*n* = 5). Tick marks indicate censored observations. Time was calculated from epcoritamab initiation. Survival comparisons were performed using the log-rank test and should be interpreted as exploratory because of the small sample size. The median follow-up time estimated by the reverse Kaplan–Meier method was 431.0 days in the LBCL cohort and 431.5 days in the FL cohort.

Overall survival (OS) and progression-free survival (PFS) from epcoritamab initiation are shown in Figure 1. In the LBCL cohort, the median OS was 431 days. Because the Kaplan–Meier-estimated median PFS was considered unstable and potentially misleading due to the small sample size and substantial censoring before later events, the median PFS value was not emphasized. PFS was therefore interpreted descriptively together with the Kaplan–Meier curve, number-at-risk table, and individual clinical courses (Figure 1A,B). Both the median OS and median PFS were 431.5 days in the R/R FL cohort (Figure 1A,B). The median follow-up time estimated by the reverse Kaplan–Meier method was 431.0 days in the LBCL cohort and 431.5 days in the FL cohort. In an exploratory analysis of the R/R LBCL cohort stratified according to the number of prior treatment lines, patients who had received ≤3 prior lines (*n* = 12) showed longer OS than those who had received ≥4 prior lines (*n* = 5) by the log-rank test (*p* = 0.0140). Median OS was not reached in the ≤3-line group and was 229 days in the ≥4-line group (Figure 1C). Similarly, PFS was longer in patients who had received ≤3 prior lines than in those who had received ≥4 prior lines (*p* = 0.0072). Median PFS was not reached in the ≤3-line group and was 174 days in the ≥4-line group (Figure 1D). Because of the small sample size and sparse events, these comparisons should be interpreted as exploratory. Among the responders, the observed duration of response ranged from 28 to 786 days in the LBCL cohort and from 113 to 271 days in the FL cohort. The median observed response duration was 98 days in LBCL and 225 days in FL. However, because the number of response-failure events was small and most responders were censored without documented progression, the Kaplan–Meier-estimated median duration of response (DOR) was not reached in either cohort. Formal time-to-next treatment (TTNT) analysis was not performed because TTNT events were rare: only two patients developed progressive disease, and only one of them received subsequent salvage therapy during the observation period.

**Figure 2:**
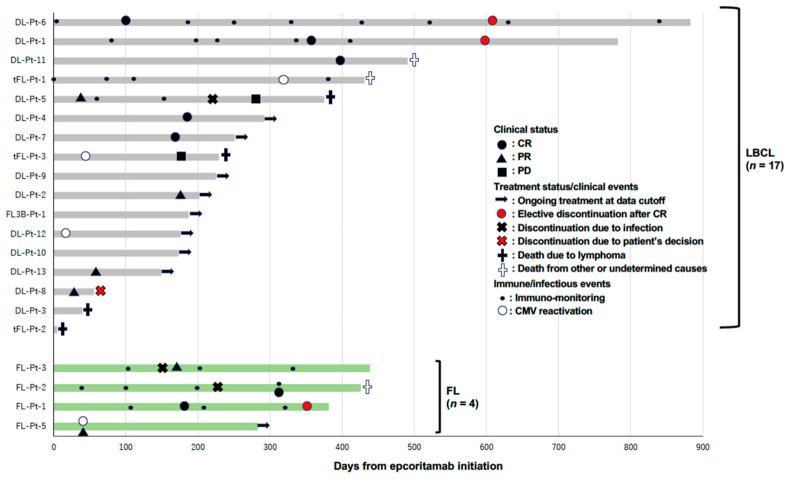
Swimmer plot showing the clinical courses of patients treated with epcoritamab. Each horizontal bar represents the observation period from epcoritamab initiation to treatment discontinuation, death, or last follow-up. Symbols indicate the first documentation of complete response, partial response, progressive disease or relapse, immune monitoring time points, infection-related events, CMV reactivation, treatment discontinuation, and death. Arrows indicate patients who remained on epcoritamab treatment at the data cutoff. CMV, cytomegalovirus; CR, complete response; PD, progressive disease; PR, partial response; tFL, DLBCL transformed from follicular lymphoma.

The clinical courses of all patients, including response status, treatment status, clinical events, and immune/infectious events, are summarized in the swimmer plot shown in Figure 2. Epcoritamab was electively discontinued after confirmation of CR in 2 patients with LBCL and 1 patient with FL, and no relapse was observed in these patients at the data cutoff. Four patients with LBCL died from lymphoma, whereas 2 patients with LBCL and 1 patient with FL died from other or undetermined causes. Epcoritamab was discontinued in 1 patient with LBCL because of herpes zoster and in 2 patients with FL because of COVID-19 pneumonia. Cytomegalovirus (CMV) reactivation occurred in 3 patients with LBCL and 1 patient with FL; however, epcoritamab treatment was continued in these cases.

### 2.4. Adverse Events Associated with Epcoritamab Treatment

Treatment-related infectious adverse events and CRS are summarized in Table 4.

Febrile neutropenia occurred in 2 patients (9.5%), both of whom had grade 3 events that resolved with granulocyte colony-stimulating factor (G-CSF) support and antibiotics. Cytomegalovirus reactivation was observed in 4 patients (19.0%), including 1 grade 1, 2 grade 2, and 1 grade 3 events, all of which were successfully managed with antiviral therapy. Grade 3 herpes zoster occurred in 1 patient and resolved after antiviral therapy. Hepatitis B virus reactivation occurred in 3 patients (14.3%), including 2 grade 2 events requiring antiviral treatment.

Hematologic toxicities were also observed during epcoritamab treatment. Treatment-emergent neutropenia occurred in 15 patients, thrombocytopenia in 16 patients, and anemia in 20 patients; grade ≥ 3 events were observed in 12, 2, and 2 patients, respectively. These events were managed with supportive care, including G-CSF support and transfusion as clinically indicated.

Hypogammaglobulinemia (immunoglobulin G < 500 mg/dL) was present at baseline or developed during treatment in 12 patients. Immunoglobulin replacement was administered in 8 patients according to clinical judgment, particularly in the setting of recurrent or severe infection. No patient discontinued epcoritamab solely because of non-infectious hematologic toxicity. Because this was a retrospective cohort, systematic attribution and longitudinal grading of all hematologic and immunoglobulin abnormalities were limited; this point has been added to the limitations.

Other infectious complications included Aspergillus antigen positivity (*n* = 1) and COVID-19 pneumonia (*n* = 2).

CRS occurred in 12 patients (57.1%); however, all events were grade 1, and no patient developed ICANS.

### 2.5. A Case of Clinically and Radiologically Suspected Central Nervous System (CNS) Relapse of LBCL Achieving Radiological Complete Remission with Epcoritamab

A 67-year-old man initially presented in August 2017 with headache, right thigh pain, and diplopia. Diagnostic workup at another hospital revealed intra-abdominal lymphadenopathy and tumor involvement of the adrenal gland, lumbar vertebrae, and cauda equina. Biopsy of an intra-abdominal lymph node established a diagnosis of DLBCL, not otherwise specified (NOS). Although no radiologically apparent central nervous system (CNS) lesion was identified at diagnosis, the presence of diplopia led to a clinical diagnosis of CNS involvement. The patient received six cycles of R-CHOP plus intrathecal chemotherapy, followed by six cycles of rituximab plus high-dose methotrexate, and achieved CR.

In September 2022, he developed impaired movement of the left upper extremity. Brain magnetic resonance imaging (MRI) demonstrated an approximately 2 cm contrast-enhanced subcortical frontal lesion with surrounding edema. Although histological confirmation was not obtained, the lesion was clinically and radiologically suspected to represent CNS relapse of DLBCL. He underwent whole-brain radiotherapy followed by stereotactic radiotherapy to the isolated right frontal lesion, resulting in CR, after which tirabrutinib was initiated.

**Figure 3:**
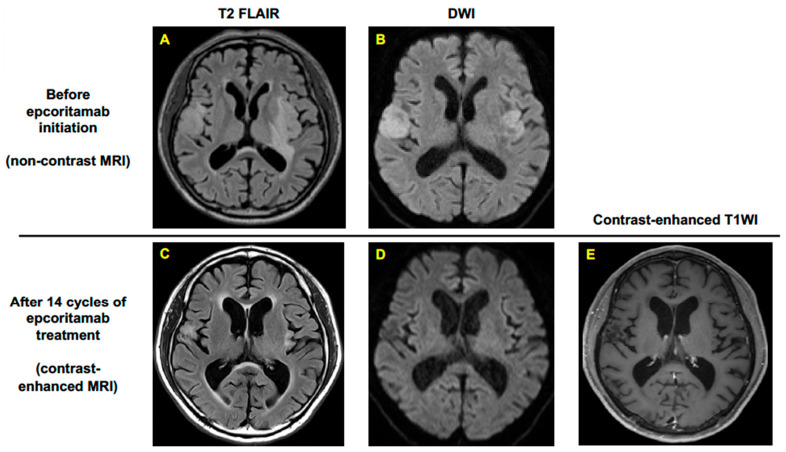
Brain magnetic resonance imaging before initiation of epcoritamab and after 14 cycles of treatment in a patient with clinically and radiologically suspected CNS relapse of DLBCL. Pretreatment non-contrast brain MRI demonstrated multiple intracranial lesions on T2 FLAIR imaging (**A**) and diffusion-weighted imaging (**B**). After 14 cycles of epcoritamab treatment, follow-up MRI showed resolution of the previously identified lesions and surrounding edema on T2 FLAIR imaging (**C**) and diffusion-weighted imaging (**D**). Post-treatment contrast-enhanced T1-weighted imaging (**E**) showed no obvious enhancing residual lesion, consistent with radiological CR. Because only non-contrast MRI was performed immediately before epcoritamab initiation, matched pre- and post-treatment contrast-enhanced T1-weighted images were not available. Representative axial images at approximately corresponding anatomical levels are shown. CNS, central nervous system; DLBCL, diffuse large B-cell lymphoma; DWI, diffusion-weighted imaging; FLAIR, fluid-attenuated inversion recovery; MRI, magnetic resonance imaging.

In June 2024, the patient again developed neurological symptoms, including general fatigue, ataxia, and drooling. Brain imaging revealed multiple recurrent lesions involving the right temporal lobe, left insular region, right frontoparietal region, and left parietal lobe. Although biopsy was not performed, these findings were clinically and radiologically suspected to represent a second CNS relapse of DLBCL. Epcoritamab therapy was initiated on 9 July 2024. Tumor shrinkage was already evident after the first cycle, with concurrent clinical improvement. After 14 cycles, MRI demonstrated complete resolution of the lesions and surrounding edema, consistent with radiological CR (Figure 3). Because only non-contrast MRI was performed immediately before epcoritamab initiation, direct comparison of pre- and post-treatment contrast-enhanced T1-weighted images was not possible. Treatment was continued through 18 cycles. In November 2025, however, the patient died suddenly after losing consciousness while bathing in a hot spring following mountain climbing. The exact cause of death remained undetermined. Although heat-related illness was clinically suspected, sudden cardiac events, occult relapse, and treatment-related complications could not be completely excluded because no autopsy was performed.

### 2.6. Longitudinal CTL Dynamics and TCR Vβ Repertoire Remodeling in Three Patients in Whom Epcoritamab Was Electively Discontinued After Complete Remission

**Figure 4:**
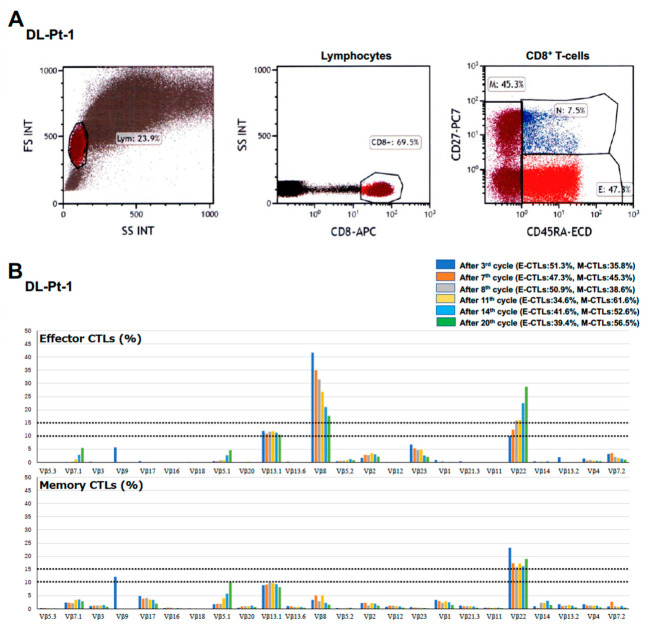

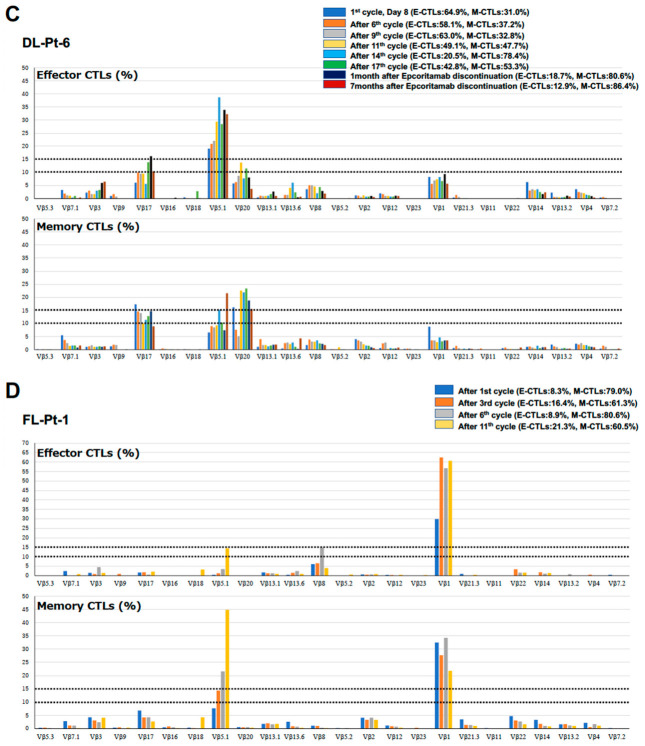
Longitudinal remodeling of cytotoxic T lymphocyte subsets and T-cell receptor Vβ repertoire in three patients who discontinued epcoritamab after achieving complete remission. (**A**) Representative flow-cytometric gating strategy for CTL subset analysis in DL-Pt-1. Peripheral blood lymphocytes were identified by FSC/SSC gating, followed by selection of CD8+ T-cells and subdivision into naïve, effector and memory CTL subsets according to CD27 and CD45RA expression. The representative plots shown were obtained after the seventh cycle of epcoritamab treatment. (**B**) Longitudinal TCR Vβ repertoire patterns in DL-Pt-1. A markedly expanded Vβ8-positive effector CTL population, expanded Vβ13.1- and Vβ22-positive effector CTL populations, and a markedly expanded Vβ22-positive memory CTL population were observed. Total effector CTLs predominated during the early phase of treatment, whereas total memory CTLs became predominant after cycle 11 and remained so through cycle 20. (**C**) Longitudinal TCR Vβ repertoire patterns in DL-Pt-6. A markedly expanded Vβ5.1-positive effector CTL population was detected from day 8 of cycle 1 onward. A markedly expanded Vβ20-positive memory CTL population was initially identified, decreased transiently, and then re-emerged after cycle 11. Total effector CTLs predominated early, but total memory CTLs became predominant later in the course and remained so after discontinuation of epcoritamab. (**D**) Longitudinal TCR Vβ repertoire patterns in FL-Pt-1. Markedly expanded Vβ1-positive effector and memory CTL populations were observed, together with progressive increase in Vβ5.1-positive effector and memory CTL populations. Total memory CTLs predominated over total effector CTLs from the first evaluable time point and remained dominant throughout the observation period. Abbreviations: CTL, cytotoxic T lymphocyte; E-CTLs, effector cytotoxic T lymphocytes; FSC, forward scatter; M-CTLs, memory cytotoxic T lymphocytes; SSC, side scatter.

Longitudinal changes in CTL subsets and TCR Vβ repertoire were analyzed in two patients with R/R LBCL and one patient with R/R FL who achieved radiological CR and subsequently discontinued epcoritamab without immediate relapse. Two patients with R/R LBCL discontinued epcoritamab after 21 cycles, whereas one patient with R/R FL discontinued treatment after 12 cycles. As shown in Figure 4A, peripheral blood lymphocytes were first identified by forward scatter (FSC) and side scatter (SSC) gating, after which CD8^+^ T-cells were subdivided into naïve, effector, and memory CTL fractions according to CD27 and CD45RA expression. TCR Vβ repertoire skewing within CTL subsets was then assessed using the IOTest Beta Mark TCR Vβ Repertoire Kit (Figure 4B–D).

DL-Pt-1 was a 62-year-old man with refractory DLBCL of germinal center B-cell (GCB) type who had received two prior lines of therapy. After three cycles of epcoritamab, a markedly expanded Vβ8-positive effector CTL population, expanded Vβ13.1- and Vβ22-positive effector CTL populations, and a markedly expanded Vβ22-positive memory CTL population were detected. Among these, the Vβ13.1-positive effector CTL population remained relatively stable throughout the clinical course, whereas the proportion of the Vβ8-positive effector CTL population gradually declined over time and that of the Vβ22-positive effector CTL population progressively increased. The Vβ22-positive memory CTL population remained relatively constant throughout the observation period. Notably, total effector CTLs predominated during the early phase of treatment, but this relationship reversed after cycle 11, after which total memory CTLs remained predominant through cycle 20. At the end of cycle 20, computed tomography (CT) confirmed CR, lactate dehydrogenase (LDH) was 229 U/L, and soluble interleukin-2 receptor (sIL-2R) was 755 U/mL. In light of concordant radiological, serological, and immunological improvement, epcoritamab was discontinued after 21 cycles following discussion with the patient and with his consent. No evidence of relapse has been observed during 4 months of follow-up after treatment discontinuation.

DL-Pt-6 was an 86-year-old man with refractory high-grade B-cell lymphoma (HGBCL) who had received three prior lines of therapy. A markedly expanded Vβ5.1-positive effector CTL population was already detectable on day 8 of cycle 1, at a time when epcoritamab had been administered only once. At this time point, total effector CTLs exceeded total memory CTLs. The Vβ5.1-positive effector CTL population remained detectable throughout the treatment course as a markedly expanded Vβ-family CTL population. In parallel, a markedly expanded Vβ20-positive memory CTL population was initially identified on day 8 of cycle 1, declined transiently, and then reemerged as a markedly expanded memory population after cycle 11. Strikingly, the balance between total effector and memory CTLs approached equivalence after cycle 11 and subsequently shifted toward clear predominance of memory CTLs. At the end of cycle 20, CT confirmed CR, LDH was 321 U/L, and sIL-2R was 558 U/mL. Because radiological CR, favorable serological findings, and supportive exploratory immune monitoring findings had all been documented, epcoritamab was discontinued after 21 cycles with the patient’s consent. No relapse has been detected during 8 months of follow-up. Moreover, at 1 and 7 months after discontinuation, the Vβ5.1-positive effector CTL population and the Vβ20-positive memory CTL population remained detectable as markedly expanded Vβ-family CTL populations, while total memory CTLs remained consistently elevated at >80%, suggesting persistence of a memory-oriented CTL phenotype after treatment cessation.

FL-Pt-1 was a 69-year-old woman with relapsed FL who had received three prior lines of therapy. After the first cycle of epcoritamab, markedly expanded Vβ1-positive effector and memory CTL populations were identified. These Vβ1-positive CTL populations persisted through cycle 11. In addition, a Vβ5.1-positive effector CTL population progressively increased over time and reached 14.4% after 11 cycles. A Vβ5.1-positive memory CTL population also increased longitudinally and was recognized as a markedly expanded Vβ-family CTL population from cycle 6 onward. In contrast to the two R/R LBCL cases, this patient exhibited a marked predominance of total memory CTLs over total effector CTLs from the earliest evaluable time point after cycle 1, and this pattern remained stable through cycle 11. At the end of cycle 11, CT confirmed CR, LDH was 256 U/L, and sIL-2R was 457 U/mL. Given the convergence of radiological CR, favorable serological findings, and sustained Vβ-skewed CTL remodeling, epcoritamab was discontinued after 12 cycles following discussion with the patient and with her consent.

Taken together, these three cases shared several immunological features: emergence and persistence of Vβ-skewed CTL populations, progressive enrichment of memory CTLs during treatment, and maintenance of remission after elective discontinuation of epcoritamab. These findings should be interpreted as exploratory phenotypic associations rather than evidence of lymphoma-specific or functionally validated CTL responses.

### 2.7. Longitudinal CTL Dynamics and TCR Vβ Repertoire Patterns in Four Patients Who Discontinued Epcoritamab Because of Infection

**Figure 5:**
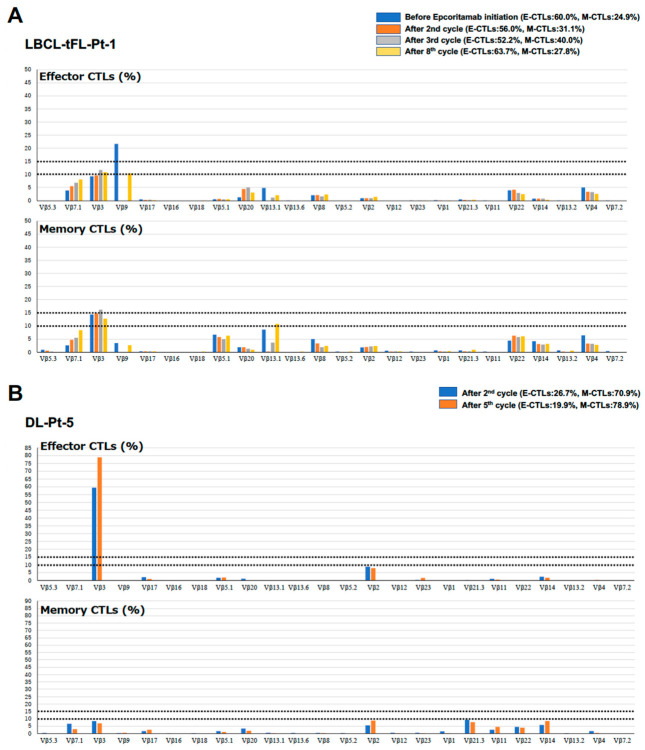

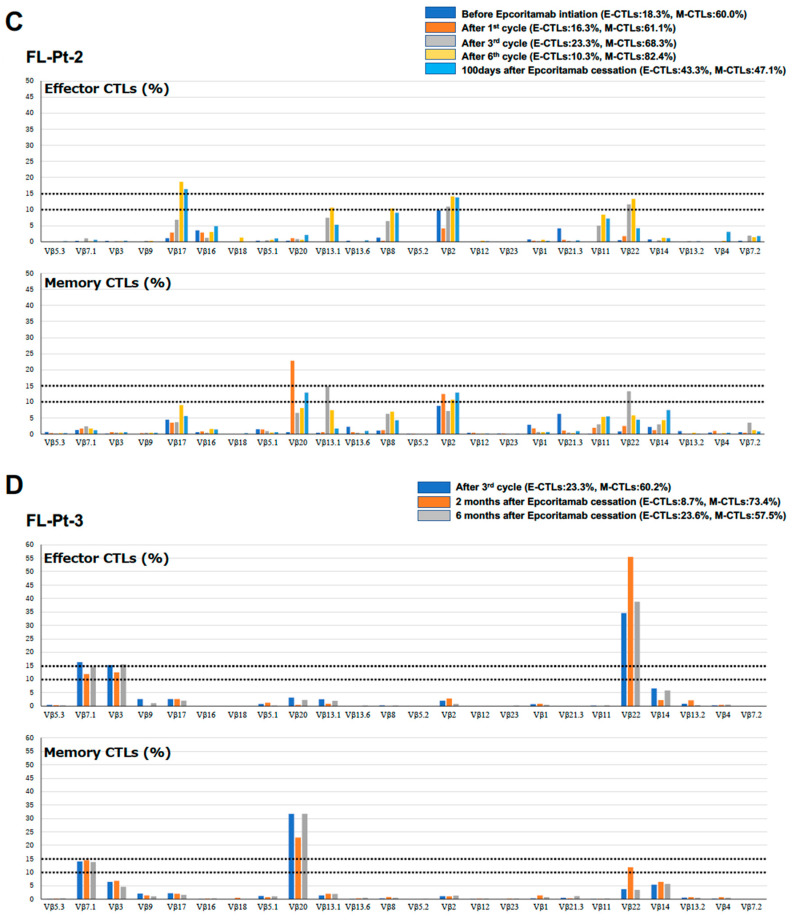
Longitudinal remodeling of cytotoxic T lymphocyte subsets and T-cell receptor Vβ repertoire in four patients who discontinued epcoritamab because of infectious complications. (**A**) Longitudinal TCR Vβ repertoire patterns in LBCL-tFL-Pt-1, a patient with LBCL transformed from FL. Expanded Vβ3-positive effector and memory CTL populations were observed, but no markedly expanded effector or memory Vβ-family CTL populations emerged during treatment. Total effector CTLs remained more abundant than total memory CTLs throughout the clinical course. (**B**) Longitudinal TCR Vβ repertoire patterns in DL-Pt-5. A markedly expanded Vβ3-positive effector CTL population was detected after cycles 2 and 5. Total memory CTLs exceeded total effector CTLs at both time points, but no expanded memory Vβ-family CTL population was observed. (**C**) Longitudinal TCR Vβ repertoire patterns in FL-Pt-2. A markedly expanded Vβ17-positive effector CTL population emerged after six cycles of epcoritamab, whereas no expanded memory Vβ-family CTL population was identified. Total memory CTLs exceeded total effector CTLs throughout treatment and reached >80% after cycle 6, but declined after treatment discontinuation. (**D**) Longitudinal TCR Vβ repertoire patterns in FL-Pt-3. Markedly expanded Vβ22-, Vβ7.1-, and Vβ3-positive effector CTL populations, together with a markedly expanded Vβ20-positive memory CTL population, were detected after cycle 3. Total memory CTLs remained the predominant fraction both during treatment and after discontinuation of epcoritamab. Abbreviations: CTL, cytotoxic T lymphocyte; E-CTLs, effector cytotoxic T lymphocytes; FSC, forward scatter; M-CTLs, memory cytotoxic T lymphocytes; SSC, side scatter.

Longitudinal changes in CTL subsets and TCR Vβ repertoire in four patients who were forced to discontinue epcoritamab because of infectious complications are shown in Figure 5. These included two patients with R/R LBCL and two R/R FL.

LBCL-tFL-Pt-1 was a 75-year-old woman with refractory LBCL transformed from FL who had received eight prior lines of therapy before epcoritamab. Before initiation of epcoritamab, an expanded Vβ3-positive memory CTL population was detectable, whereas no other clearly expanded Vβ-family CTL populations were identified. This pattern remained largely unchanged during treatment. Even after eight cycles, only expanded Vβ3-positive effector and memory CTL populations were observed, without the emergence of markedly expanded effector or memory Vβ-family CTL populations. In addition, total effector CTLs remained more abundant than total memory CTLs throughout the clinical course (Figure 5A). This patient also had lymphoma involvement of the right main bronchus and died suddenly 2 weeks after completion of cycle 8 because of aspiration pneumonia and airway obstruction by sputum. The best response was stable disease (SD).

DL-Pt-5 was a 74-year-old man with refractory HGBCL who had received three prior lines of therapy before epcoritamab. After two cycles of epcoritamab, a markedly expanded Vβ3-positive effector CTL population was identified, and this population remained detectable as a markedly expanded effector CTL population after five cycles. At both time points, the proportion of total memory CTLs exceeded that of total effector CTLs. However, no expanded memory Vβ-family CTL population was detected at either evaluation point (Figure 5B). After seven cycles, the patient developed herpes zoster and epcoritamab was discontinued. Although the treatment response at discontinuation was PR, the disease subsequently progressed rapidly. The patient declined further aggressive treatment, and palliative care was selected.

FL-Pt-2 was a 73-year-old man with refractory FL who had received six prior lines of therapy before epcoritamab. Before treatment initiation, no clearly expanded Vβ-family CTL populations were observed, although total memory CTLs already exceeded total effector CTLs. After six cycles, a markedly expanded Vβ17-positive effector CTL population emerged; however, no sufficiently expanded memory Vβ-family CTL population was identified at that time. In contrast, the proportion of total memory CTLs increased to more than 80% (Figure 5C). Epcoritamab was discontinued after six cycles because of COVID-19 infection, at which point the patient had achieved CR. At 100 days after discontinuation, the Vβ17-positive effector CTL population remained detectable as a markedly expanded Vβ-family CTL population, but the proportion of total memory CTLs had decreased by approximately half, to the 40% range. Nevertheless, CR remained maintained at approximately 6 months after treatment discontinuation.

FL-Pt-3 was a 73-year-old woman with refractory FL who had received three prior lines of therapy before epcoritamab. After three cycles of treatment, markedly expanded Vβ22-, Vβ7.1-, and Vβ3-positive effector CTL populations were identified, together with a markedly expanded Vβ20-positive memory CTL population. At the same time, total memory CTLs accounted for approximately 60% of CTLs and represented the major fraction (Figure 5D). Epcoritamab was discontinued after five cycles because of COVID-19 infection, at which time the treatment response was PR. At both 2 and 6 months after discontinuation, the Vβ22-positive effector CTL population and the Vβ20-positive memory CTL population remained detectable as markedly expanded Vβ-family CTL populations. The proportion of total memory CTLs first increased to 73.4% at 2 months after discontinuation and then declined to 57.5% at 6 months, although it remained the predominant fraction. Clinically, PR was maintained for approximately 8 months after treatment discontinuation.

Collectively, these four cases suggest that discontinuation of epcoritamab because of infection does not necessarily result in immediate loss of disease control. However, the immunological patterns appeared heterogeneous. These observations suggest that infection-related discontinuation was associated with heterogeneous immune monitoring patterns, and that persistence of memory-predominant Vβ-skewed CTL populations may warrant further evaluation as a potential correlate of ongoing clinical response.

## 3. Discussion

In the present study, we evaluated the clinical efficacy, safety, and immunological correlates of epcoritamab therapy in patients with R/R LBCL and R/R FL. Several findings warrant particular emphasis. First, epcoritamab demonstrated clinically meaningful activity in both R/R LBCL and R/R FL in a real-world setting, including CR in selected patients. Second, epcoritamab monotherapy was associated with radiological complete remission in a patient with clinically and radiologically suspected CNS relapse of LBCL, suggesting that its antitumor effects may extend beyond direct tumor-cell engagement, although alternative explanations cannot be excluded. Third, serial immune monitoring revealed that favorable clinical outcomes were associated not only with radiological and serological improvement, but also with the emergence and persistence of Vβ-skewed CTL populations, particularly within the memory CTL compartment. Collectively, these findings support the hypothesis that the therapeutic benefit of epcoritamab may be associated with longitudinal CTL remodeling, although lymphoma specificity and functional cytotoxicity were not demonstrated [1,4,10].

Recent progress in the treatment of refractory B-cell lymphomas has clearly established the central importance of T-cell activation. Both CAR-T therapy and CD3×CD20 bispecific antibodies have improved outcomes in patient populations that historically had very limited therapeutic options. As recently reviewed, CD3×CD20 bispecific antibodies are now regarded as a major therapeutic class in R/R B-cell lymphomas, with epcoritamab, glofitamab, mosunetuzumab, and odronextamab demonstrating clinically meaningful activity across different disease settings [11,12,13,14,15]. At the same time, these agents differ from CAR-T therapy in important practical respects: CAR-T therapy is highly active, but requires specialized centers, manufacturing time, and management of substantial toxicities, whereas bispecific antibodies are more readily deployable in routine practice and generally have a more manageable safety profile [5,11]. This distinction is particularly relevant in elderly or frail patients, who constituted a substantial proportion of our cohort. Thus, although CAR-T therapy remains indispensable in selected settings, bispecific antibodies are likely to assume an increasingly central role in real-world management.

The baseline characteristics of our cohort should be considered when interpreting the clinical outcomes. The LBCL cohort included a substantial proportion of elderly patients, patients with poor performance status, advanced-stage disease, extranodal involvement, high IPI scores, and refractoriness to the immediately preceding therapy. In addition, none of the patients had received prior CAR-T-cell therapy, and all retained CD20 expression. Therefore, the present cohort reflects a high-risk but CAR-T-naive real-world population, and the findings may not be directly generalizable to post-CAR-T or CD20-negative disease settings.

Our clinical outcomes should also be interpreted in the context of the pivotal epcoritamab studies. In EPCORE NHL-1, epcoritamab produced durable responses in both LBCL and FL, with the greatest long-term benefit observed in patients who achieved CR [1,2,3,4]. This pattern is highly consistent with our cohort, in which durable disease control was also concentrated in patients who achieved CR. However, the present study extends those observations by adding longitudinal immune profiling. Whereas prior clinical studies established the importance of depth of response, our data suggest that durable remission may be accompanied by a distinctive immunological state characterized by Vβ-family over-representation within CTL subsets and enrichment of memory CTLs. In this sense, our findings do not contradict the pivotal efficacy data, but rather provide a possible immunological framework to explain why only a subset of patients derives sustained benefit.

One of the most notable observations in the present study was the radiological CR achieved with epcoritamab monotherapy in a patient with clinically and radiologically suspected CNS relapse of LBCL. Secondary CNS lymphoma remains a clinically challenging condition with historically poor outcomes, and optimal treatment strategies continue to evolve [21]. In PCNSL and SCNSL, CAR-T therapy has shown clinically meaningful antitumor activity, including expansion and trafficking of CAR-T cells into the CNS and favorable outcomes in contemporary clinical series [22,23]. By contrast, the role of bispecific antibodies in CNS lymphoma has remained less certain because of concern regarding blood–brain barrier penetration. However, recent translational work with glofitamab demonstrated that a CD3×CD20 bispecific antibody can penetrate the blood–brain barrier, stimulate immune-cell infiltration of CNS tumors, and induce clinical responses in SCNSL [24]. Although this was not an epcoritamab study, it provides important biological support for the plausibility of our observation.

From a mechanistic standpoint, our CNS case, although histological confirmation was not available, suggests that the antitumor activity of epcoritamab may not be explained solely by direct local immune synapse formation within the CNS lesion itself. Rather, a biologically plausible interpretation is that epcoritamab may have been associated with systemic immune remodeling in the peripheral compartment. However, because immune monitoring was not performed in this patient and neither CNS penetration of epcoritamab nor CTL trafficking was directly demonstrated, this interpretation remains speculative. If so, epcoritamab may exert dual antitumor effects: an immediate effect through redirected T-cell killing and a secondary effect through amplification of systemic immune remodeling associated with clinical disease control. This interpretation is concordant with our previous observations in B-cell ALL, ATLL, and CML, in which therapeutic efficacy was positively associated with activation of effector and memory CTLs [16,17,18,19,20]. Thus, although direct evidence of CNS trafficking was not available, the present CNS case may represent a clinically important extension of the broader concept that durable tumor control may be associated with longitudinal CTL remodeling, although this remains hypothesis-generating [24].

Another important finding was the significantly superior OS observed in patients with R/R LBCL who had received three or fewer prior lines of therapy compared with those treated later. Although this analysis was limited by sample size, it is biologically plausible and aligns with recent biomarker and review literature on CD3×CD20 bispecific antibodies. Emerging data suggest that response to bispecific antibodies may be influenced by tumor-intrinsic immune evasion, host T-cell fitness, and the broader immune microenvironment [10,11]. As lymphoma evolves under repeated therapeutic pressure, tumor cells may acquire more pronounced immune-evasive phenotypes, while host T-cell competence progressively deteriorates. Under such conditions, even a potent T-cell-engaging agent may be operating against both a more resistant tumor and a less fit immune system. Our observation that late-line treatment was associated with inferior survival is therefore consistent with the broader literature and supports the view that epcoritamab may be more effective when introduced earlier, before cumulative immune attrition becomes profound [10,11]. The comparison should be interpreted cautiously, as the small sample size precluded adjustment for potential confounders such as histologic heterogeneity, age, and prior treatment exposure.

This point has practical therapeutic implications. Contemporary reviews increasingly discuss earlier integration of bispecific antibodies and combination strategies rather than reserving these agents exclusively for late salvage [11]. In principle, biologically high-risk B-cell lymphomas might be managed with an initial rituximab-containing chemoimmunotherapy phase for tumor debulking, followed by bispecific antibody therapy to consolidate remission through immune-mediated control. Such a strategy could both reduce cumulative cytotoxic chemotherapy exposure and exploit a more intact host T-cell compartment. Our data do not prove this approach, but they are directionally consistent with that line of thinking and support further exploration of earlier incorporation of epcoritamab-based therapy. This concept is also supported by emerging epcoritamab-based combination data, including epcoritamab plus gemcitabine and oxaliplatin in transplant-ineligible R/R DLBCL, which demonstrated deep and durable responses in an earlier-line salvage setting [25].

A central and distinctive aspect of the present study was serial assessment of CTL subsets and TCR Vβ repertoire. At present, the optimal duration of epcoritamab therapy remains undefined, and there are no established biomarkers to indicate when treatment discontinuation may be considered safe. Indeed, the pivotal epcoritamab studies and their long-term follow-up largely used treatment continuation until progression or unacceptable toxicity, rather than biology-guided elective discontinuation [1,2,3,4]. This is an important unmet clinical issue, because some patients appear to achieve deep and durable remission, yet evidence-based stopping criteria are lacking. Against this background, our study is exploratory but clinically relevant in that treatment discontinuation in selected cases was guided not only by imaging and conventional serological markers such as LDH and sIL-2R, but also by immunological parameters, particularly TCR Vβ repertoire skewing and the balance between effector and memory CTL compartments.

In the two R/R LBCL cases and one R/R FL case in whom epcoritamab was electively discontinued, expanded Vβ-family CTL populations were detected, and, importantly, total memory CTLs exceeded total effector CTLs at the time of discontinuation. This shift is biologically meaningful. If tumor burden has been markedly reduced or eliminated, contraction of the effector compartment and emergence of a memory-dominant immune state would be expected. In this sense, the transition from effector predominance to memory predominance may represent an immunological signature of successful tumor control and post-treatment immune consolidation. Our data therefore extend the current biomarker discussion beyond simple response-associated correlates and suggest that longitudinal immune architecture may help define when a patient has entered a more stable immune state associated with clinical disease control [10]. However, these observations should be interpreted with caution given the limited sample size and the exploratory nature of the immune analyses, and therefore require validation in larger prospective cohorts.

This interpretation is supported, in a hypothesis-generating manner, by the persistence of expanded Vβ-family CTL populations after treatment cessation. In the two R/R LBCL cases in whom epcoritamab was electively discontinued after 21 cycles, memory CTL predominance was maintained at 4 and 8 months after discontinuation, respectively. Moreover, in one of these cases, expanded effector and memory Vβ-family CTL populations remained detectable after treatment cessation, suggesting that these populations may represent a phenotypic correlate of ongoing immune surveillance, although lymphoma specificity was not demonstrated. In this context, persistence of markedly expanded memory Vβ-family CTL populations may represent a phenotypic correlate of durable disease control, although functional activity and antigen specificity were not demonstrated. This interpretation is highly concordant with our previous work in chronic-phase CML, in which safe treatment-free remission after tyrosine kinase inhibitor discontinuation was associated not only with prolonged deep molecular response but also with enhanced antitumor immunity, including expanded effector and/or memory CTL populations and predominance of total memory CTLs over total effector CTLs [20]. By analogy, the present findings suggest that discontinuation of epcoritamab might be approached more safely when radiological CR, favorable serological markers, and sustained memory-oriented CTL activation are all present. Among these factors, the presence of markedly expanded memory Vβ-family CTL populations may be of particular importance as an indicator of durable immune surveillance. These findings require validation in larger prospective cohorts before clinical application.

The contrast between the elective discontinuation cases and the infection-related discontinuation cases further supports this interpretation. In the patient with transformed LBCL after eight prior lines of therapy who exhibited treatment resistance, sustained markedly expanded effector or memory Vβ-family CTL populations were never established, and total effector CTLs remained predominant throughout the clinical course. This pattern is consistent with current concepts in bispecific antibody resistance, in which absence of robust T-cell fitness and failure to establish effective immune remodeling may limit durable benefit. The case of DL-Pt-5 is also instructive. Although a markedly expanded effector Vβ-family CTL population emerged after five cycles and total memory CTLs accounted for nearly 80% of the CTL compartment, no markedly expanded memory Vβ-family CTL population was identified. At the time of treatment interruption due to herpes zoster, the best response was only PR, and the disease subsequently progressed rapidly. This suggests, first, that discontinuation in the setting of PR may carry a substantial risk of relapse or progression and, second, that effector activation alone may be insufficient to confer durable disease control in the absence of a robust memory CTL component. Immunologically, this case implies that establishment of markedly expanded memory Vβ-family CTL populations may be more informative than transient effector activation when considering the likelihood of sustained remission.

The FL cases provide additional nuance. In FL-Pt-2, a markedly expanded effector Vβ-family CTL population emerged after six cycles, and total memory CTLs accounted for more than 80% of the CTL compartment at that time; however, no markedly expanded memory Vβ-family CTL population was identified. Although CR was maintained for approximately 6 months after discontinuation due to COVID-19, the proportion of total memory CTLs had fallen to the 40% range by 100 days after stopping treatment. This suggests that even when short- to intermediate-term disease control is preserved, the immunological basis for that control may progressively weaken after treatment interruption. FL-Pt-3 appeared more favorable immunologically, as both markedly expanded effector and memory Vβ-family CTL populations were detected early and persisted for several months after discontinuation. Even in this case, however, the proportion of total memory CTLs gradually declined over time after treatment cessation. These observations suggest that persistence of expanded Vβ-family CTL populations and memory predominance may support continued disease control after treatment interruption, but that gradual contraction of the memory compartment could herald future loss of immune protection. Thus, even in patients who remain clinically stable after discontinuation for non-oncologic reasons, close radiological and immunological follow-up appears warranted.

Infection-related immune remodeling is an important potential confounder in this study. Viral infections and reactivations, including CMV reactivation, HBV reactivation, herpes zoster, and COVID-19 pneumonia, may independently reshape effector and memory T-cell compartments and may also influence TCR Vβ repertoire skewing. In the present cohort, the timing of infection-related events and immune monitoring is summarized in the swimmer plot (Figure 2). Nevertheless, because this was a retrospective study and immune monitoring was performed at clinically available time points rather than according to a predefined schedule, the relative contributions of epcoritamab-associated immune remodeling and infection-induced reactive T-cell changes could not be fully separated.

The present study also has implications for the unresolved question of treatment duration. At present, there is no consensus regarding how long epcoritamab should be continued, nor are there validated criteria for safe discontinuation. Existing monotherapy studies have largely been designed around treatment until progression or unacceptable toxicity, whereas fixed-duration strategies remain under active development rather than established standard practice [1,2,3,4,11]. Notably, fixed-duration epcoritamab-based combination therapy, such as epcoritamab plus rituximab and lenalidomide in R/R FL, has shown favorable outcomes, further underscoring the clinical importance of defining biologically informed discontinuation criteria [26]. Against this background, our data suggest that treatment duration should not be considered in isolation. Rather, duration should be interpreted in the context of disease status and immune remodeling. In the present cohort, patients who discontinued treatment without CR or without establishment of a favorable immunological state appeared to be at higher risk of subsequent relapse or progression. By contrast, elective discontinuation after 12 or 21 cycles in patients who had achieved CR and exhibited robust CTL activation, particularly within the memory compartment, was followed by continued remission. These observations raise the hypothesis that a certain period of continued treatment may be required in some patients to allow immune remodeling; however, the present data are insufficient to define a minimum number of cycles. More broadly, the current findings argue that the optimal duration of epcoritamab may need to be individualized biologically rather than fixed uniformly, with serial immune monitoring requiring prospective validation before it can be used to inform treatment continuation or discontinuation.

This study has several important limitations. First, this was a single-center retrospective study with a small sample size and heterogenous histological subtypes and prior treatment histories. Second, immune monitoring was performed only in selected patients and at clinically available time points, rather than according to a predefined prospective sampling schedule, introducing potential selection bias. Third, the CTL phenotyping panel was limited to CD8, CD27, and CD45RA; therefore, central memory and effector memory subsets were not separately distinguished, and functional or exhaustion markers, including PD-1, TIM-3, LAG-3, TIGIT, TOX, EOMES, T-bet, Ki-67, granzyme B, perforin, and intracellular interferon gamma, were not assessed. Fourth, CD3 was not included in the TCR Vβ panel, and therefore minor contamination by non-conventional CD8-positive populations cannot be completely excluded. Fifth, TCR sequencing was not performed; consequently, CDR3-defined clonality, antigen specificity, and lymphoma reactivity of the Vβ-skewed CTL populations could not be established. Sixth, the TCR Vβ flow-cytometric panel covered only approximately 70% of the human TCR Vβ repertoire, and family-specific reference ranges and assay coefficients of variation were not available. Seventh, no immune monitoring was performed in the patient with clinically and radiologically suspected CNS relapse. Finally, because no prospective validation cohort was available, the potential role of serial immune monitoring in treatment discontinuation should be regarded strictly as hypothesis-generating. Prior bendamustine exposure, particularly in the FL cohort, may also have influenced T-cell fitness and immune-repertoire dynamics, but its impact could not be formally assessed because of the small sample size. In addition, because a uniform operational threshold was applied across all Vβ families despite differences in their physiological baseline frequencies, this approach may overestimate expansion in inherently low-frequency Vβ families and underestimate expansion in high-frequency families.

In conclusion, the present study suggests that the antitumor activity of epcoritamab may reflect not only acute T-cell redirection through CD3×CD20-mediated immune synapse formation, but also induction of longitudinal CTL remodeling associated with clinical disease control. Earlier use of epcoritamab may be more effective than very late-line administration, likely because tumor immune escape and host immune dysfunction become more pronounced after repeated prior therapy [10,11]. Furthermore, our findings raise the possibility that treatment discontinuation might be guided not only by imaging and serological markers, but also by immune monitoring, particularly the presence of markedly expanded memory Vβ-family CTL populations and a shift toward memory CTL predominance, although this approach requires validation in larger prospective cohorts with functional and sequence-based T-cell analyses. If validated in larger prospective studies, such an approach could provide a biologically informative framework for defining optimal treatment duration and for prospectively evaluating treatment discontinuation of epcoritamab.

## 4. Materials and Methods

### 4.1. Patients

All patients included in the present study initiated epcoritamab monotherapy between December 2023 and December 2025 at Japanese Red Cross Nagasaki Genbaku Hospital. The study cohort consisted of 17 patients with R/R LBCL and 4 patients with R/R FL. Histological diagnoses were classified according to the fifth edition of the WHO Classification of Haematolymphoid Tumours [27,28].

### 4.2. Study Design

This was a non-randomized, non-blinded, single-center retrospective study approved by the Central Ethical Review Board of the Japanese Red Cross Nagasaki Genbaku Hospital (approval no. R7-835; approval date: 13 August 2025). The study was conducted in accordance with the principles of the 1964 Declaration of Helsinki and its later amendments. Consecutive patients who initiated epcoritamab at our institution between 1 December 2023 and 31 December 2025 were included. No patient who initiated epcoritamab during this period was excluded. Clinical follow-up was updated through 18 May 2026, which was used as the data cutoff for survival, response durability, treatment status, and swimmer plot analyses.

Response was assessed according to the Lugano 2014 classification. Positron emission tomography-computed tomography (PET/CT) was used whenever clinically available, and CT was used when PET/CT was not performed. Patients were considered response-evaluable if they underwent at least one imaging-based response assessment after epcoritamab initiation or had clinically evident progressive disease or death before scheduled imaging.

OS was defined as the time from epcoritamab initiation to death from any cause. PFS was defined as the time from epcoritamab initiation to documented disease progression, relapse, or death from any cause, whichever occurred first. Patients without an event were censored at the last follow-up or last disease assessment, as appropriate.

No formal predefined stopping rule was used. Decisions regarding elective discontinuation were made on a case-by-case basis according to radiological CR, favorable serological findings, and supportive immunological findings from CTL subset and TCR Vβ repertoire analyses.

### 4.3. Epcoritamab Treatment and Adverse Event Assessment

Epcoritamab was administered subcutaneously according to the approved step-up dosing schedule, consisting of priming, intermediate, and first full doses followed by subsequent full-dose administration. Premedication and prophylactic measures for cytokine release syndrome were performed according to the approved label and standard institutional practice, including corticosteroids, antipyretics, and antihistamines as clinically appropriate. CRS was graded according to CTCAE version 5.0 and managed according to institutional practice; tocilizumab was available for clinically significant CRS.

### 4.4. Analysis of CTLs and TCR Vβ Repertoire

CTL subset and TCR Vβ repertoire analyses were performed by flow cytometry. TCR Vβ repertoire skewing of CD8^+^ events was assessed using the IOTest Beta Mark TCR Vβ Repertoire Kit (Beckman Coulter, Brea, CA, USA; catalog number IM3497), according to the manufacturer’s instructions. Flow cytometric acquisition was performed using a NAVIOS EX flow cytometer (Beckman Coulter, Tokyo, Japan), and data were analyzed using Kaluza Analysis v2.2 (Beckman Coulter). The IOTest Beta Mark TCR Vβ Repertoire Kit evaluates 24 Vβ families and covers approximately 70% of the human TCR Vβ repertoire. Therefore, Vβ-skewed populations outside the antibody panel could not be detected, and the absence of a detectable expanded Vβ-family population does not exclude the presence of T-cell expansion outside the covered repertoire.

Whole-blood samples were incubated with the corresponding fluorochrome-conjugated monoclonal antibodies for 10 min at room temperature. Erythrocytes were lysed using OptiLyse C Lysing Solution (Beckman Coulter) according to the manufacturer’s instructions. After lysis, cells were washed once with phosphate-buffered saline and resuspended for acquisition. No fixation/permeabilization step was performed.

CTL subsets were defined within CD8^+^ lymphocytes according to CD27 and CD45RA expression as follows: naïve, CD8^+^CD27^+^CD45RA^+^; effector, CD8^+^CD27^−^CD45RA^+^; and memory, CD8^+^CD45RA^−^ [19,20]. For TCR Vβ repertoire analysis, lymphocytes were first gated according to forward scatter and side scatter, followed by gating on CD8^+^ events, and the frequencies of individual Vβ families were determined using the IOTest Beta Mark Vβ Repertoire Kit according to the manufacturer’s instructions. Because CD3 was not included in the TCR Vβ panel, minimal natural killer (NK) cell contamination cannot be completely excluded. However, given the absence of TCR Vβ expression in NK cells, such contamination would not result in false-positive Vβ-family over-representation signals. Frequencies of naïve, effector, and memory subsets were calculated as percentages of total CD8^+^ cells (CD8^+^ = 100%). Frequencies of individual Vβ families were calculated within each subset gate, i.e., within effector CTLs and memory CTLs, respectively.

For TCR Vβ repertoire analysis, Vβ-family over-representation was operationally categorized as follows: expanded Vβ-family CTL populations, 10–14.9%; and markedly expanded Vβ-family CTL populations, ≥15.0% of a given Vβ family. In this study, the term “CTL” refers to phenotypically defined CD8^+^ T lymphocyte subsets based on CD27/CD45RA classification and does not necessarily indicate confirmed cytotoxic function. According to the manufacturer’s reference data obtained from normal specimens, the relative representation of individual TCR Vβ families varies widely across Vβ families in CD3^+^ and CD3^+^CD8^+^ T-cell subsets. Therefore, the terms “expanded” and “markedly expanded” were used as operational descriptors of Vβ over-representation within effector and memory CTL subsets rather than as surrogates for functional activation. Definitive assessment of clonality and antigen specificity would require sequencing and/or functional assays. Accordingly, the thresholds used to define expanded and markedly expanded Vβ-family CTL populations should be interpreted as operational descriptors for standardized longitudinal comparison rather than as validated biomarker cutoffs. Details of antibodies, kits, clone names, catalog numbers, fluorochromes, and manufacturers are provided in Supplemental Table S1.

### 4.5. Statistical Analysis

All statistical analyses were performed using GraphPad Prism 9.5.1 (GraphPad Software, San Diego, CA, USA). OS and PFS were estimated using the Kaplan–Meier method. Survival time was calculated from the date of epcoritamab initiation. Patients without an event were censored at the date of last follow-up for OS and at the date of last disease assessment for PFS. Survival curves were compared using the two-sided log-rank test. A *p* value < 0.05 was considered statistically significant. Duration of response was defined among patients who achieved complete or partial response as the time from the first documentation of response to documented disease progression, relapse, or death from any cause. Patients without progression, relapse, or death were censored at the date of the last disease assessment. Because response-failure events were limited, DOR was summarized descriptively and was not shown as a separate Kaplan–Meier curve. Because of the small sample size and sparse events, survival comparisons, including analyses stratified by prior treatment lines, were regarded as exploratory. Hazard-ratio estimates from Cox models were not emphasized because of instability in sparse-data settings.

## Figures and Tables

**Table 1:** Baseline characteristics of patients treated with epcoritamab.

Characteristic	LBCL (*n* = 17)	FL (*n* = 4)
Age, years		
Median (range)	80 (53–93)	71 (63–79)
Sex		
Male, *n* (%)	10 (58.8)	2 (50.0)
Female, *n* (%)	7 (41.2)	2 (50.0)
Histological subtype, *n* (%)		
DLBCL, GCB type	2 (11.8)	—
DLBCL, ABC type	1 (5.9)	—
DLBCL, NOS	3 (17.6)	—
HGBCL	6 (35.3)	—
T-cell-rich LBCL	1 (5.9)	—
FL grade 3B	1 (5.9)	—
DLBCL transformed from FL	3 (17.6)	—
FL grade 1–2	—	4 (100)
Prior lines of therapy, *n* (%)		
≤3 lines	12 (70.6)	2 (50.0)
≥4 lines	5 (29.4)	2 (50.0)
ECOG PS, *n* (%)		
1	2 (11.8)	3 (75.0)
2	6 (35.3)	0 (0.0)
3	7 (41.2)	1 (25.0)
4	2 (11.8)	0 (0.0)
Ann Arbor stage, *n* (%)		
I	0 (0.0)	0 (0.0)
II	1 (5.9)	0 (0.0)
III	5 (29.4)	3 (75.0)
IV	11 (64.7)	1 (25.0)
Extranodal disease, *n* (%)		
Present	11 (64.7)	1 (25.0)
Absent	6 (35.3)	3 (75.0)
Bulky disease, *n* (%)		
Present	6 (35.3)	0 (0.0)
Absent	11 (64.7)	4 (100)
IPI, *n* (%)		
Low	0 (0.0)	—
Low-intermediate	1 (5.9)	—
High-intermediate	5 (29.4)	—
High	11 (64.7)	—
FLIPI, *n* (%)		
Low	—	0 (0.0)
Intermediate	—	0 (0.0)
High	—	4 (100)
MYC expression status, *n* (%)		
Positive	10 (58.8)	0 (0.0)
Negative	0 (0.0)	0 (0.0)
Unknown	7 (41.2)	4 (100)
BCL2 expression status, *n* (%)		
Positive	11 (64.7)	4 (100)
Negative	3 (17.6)	0 (0.0)
Unknown	3 (17.6)	0 (0.0)
BCL6 expression status, *n* (%)		
Positive	9 (52.9)	3 (75.0)
Negative	1 (5.9)	0 (0.0)
Unknown	7 (41.2)	1 (25.0)
Baseline LDH (U/L)		
Median (range)	230 (147–911)	180 (146–256)
Baseline sIL-2R (U/mL)		
Median (range)	831 (487–10114)	897 (611–6089)
Prior bendamustine therapy, *n* (%)		
Yes	5 (29.4)	3 (75.0)
No	12 (70.6)	1 (25.0)
Refractory to last therapy, *n* (%)		
Yes	17 (100)	3 (75.0)
No	0 (0.0)	1 (25.0)
Prior CAR-T therapy, *n* (%)		
Yes	0 (0.0)	0 (0.0)
No	17 (100)	4 (100)
CD20 expression status, *n* (%)		
Positive	17 (100)	4 (100)
Negative	0 (0.0)	0 (0.0)
Baseline serum IgG (mg/dL)		
Median (range)	718 (297–1338)	500 (191–1041)
Time from last therapy to epcoritamab initiation, days		
Median (range)	27 (12–637)	77 (24–8264)

Bulky disease was defined as a lesion measuring >7.5 cm in the longest diameter. In patients with DLBCL transformed from FL, prior lines of therapy included treatments administered for both antecedent FL and transformed DLBCL. Refractoriness to the last therapy was defined as failure to achieve complete or partial response to the immediately preceding therapy or disease progression during or after that therapy. MYC, BCL2, and BCL6 expression statuses were assessed by immunohistochemistry using available diagnostic specimens. CD20 expression was assessed by immunohistochemistry or flow cytometry when available. ABC, activated B-cell; BCL2, B-cell lymphoma 2; BCL6, B-cell lymphoma 6; CAR-T, chimeric antigen receptor T-cell; CD20, cluster of differentiation 20; DLBCL, diffuse large B-cell lymphoma; ECOG PS, Eastern Cooperative Oncology Group performance status; FL, follicular lymphoma; FLIPI, Follicular Lymphoma International Prognostic Index; GCB, germinal center B-cell; HGBCL, high-grade B-cell lymphoma; IgG, immunoglobulin G; IPI, International Prognostic Index; LBCL, large B-cell lymphoma; LDH, lactate dehydrogenase; MYC, MYC proto-oncogene; NOS, not otherwise specified; sIL-2R, soluble interleukin-2 receptor.

**Table 2:** Treatment exposure and best response.

Variable	LBCL (*n* = 17)	FL (*n* = 4)
Epcoritamab cycles received, *n*		
Median (range)	7 (1–21)	7.5 (5–12)
Ongoing treatment at data cutoff, *n* (%)	8 (47.1)	1 (25.0)
Response-evaluable patients, *n*	14	4
Best response among evaluable patients, *n/N* (%)		
Complete response	5/14 (35.7)	2/4 (50.0)
Partial response	4/14 (28.6)	2/4 (50.0)
Stable disease	3/14 (21.4)	0/4 (0.0)
Progressive disease	2/14 (14.3)	0/4 (0.0)
Not evaluable among all treated patients, *n*/*N* (%)	3/17 (17.6)	0/4 (0.0)
Overall response rate among evaluable patients, *n*/*N* (%)	9/14 (64.3)	4/4 (100)

Treatment status was assessed at the data cutoff. Response categories were calculated among response-evaluable patients. Overall response rate was defined as the proportion of patients achieving complete or partial response among response-evaluable patients. Not evaluable patients were included in the denominator for the overall treated population but excluded from response-rate calculations. FL, follicular lymphoma; LBCL, large B-cell lymphoma; ORR, overall response rate.

**Table 3:** Reasons for discontinuation of epcoritamab treatment.

Reason for Discontinuation	*n*	Disease Group
Elective discontinuation after CR	2	LBCL
Sudden death while in CR	1	LBCL
Herpes zoster during PR	1	LBCL
Death due to PD	3	LBCL
Death due to aspiration pneumonia	1	LBCL
Patient decision	1	LBCL
Elective discontinuation after CR	1	FL
COVID-19 infection while in CR	1	FL
COVID-19 infection during PR	1	FL

CR, complete response; LBCL, large B-cell lymphoma; PD, progressive disease; PR, partial response.

**Table 4:** Treatment-related infectious adverse events and cytokine release syndrome.

Adverse Event	Any Grade, *n* (%)	Grade 1, *n*	Grade 2, *n*	Grade ≥ 3, *n*
Febrile neutropenia	2 (9.5)	0	0	2
Aspergillus antigen positivity	1 (4.8)	0	1	0
Cytomegalovirus reactivation	4 (19.0)	1	2	1
Herpes zoster	1 (4.8)	0	0	1
COVID-19 pneumonia	2 (9.5)	0	0	2
Hepatitis B virus reactivation	3 (14.3)	1	2	0
Cytokine release syndrome	12 (57.1)	12	0	0

Grading was performed according to the Common Terminology Criteria for Adverse Events (CTCAE) version 5.0.

## Data Availability

The data supporting the findings of this study are available from the corresponding author upon reasonable request. The data are not publicly available because of privacy or ethical restrictions.

## Supplementary Materials

The following supporting information can be downloaded at: https://www.mdpi.com/article/10.3390/ijms27115132/s1.

## References

[1] Thieblemont C., Phillips T., Ghesquieres H., Cheah C.Y., Clausen M.R., Cunningham D., Jurczak W., Do Y.R., Gasiorowski R., Lewis D.J. (2023). Epcoritamab, a Novel, Subcutaneous CD3×CD20 Bispecific T-Cell-Engaging Antibody, in Relapsed or Refractory Large B-Cell Lymphoma: Dose Expansion in a Phase I/II Trial. J. Clin. Oncol..

[2] Thieblemont C., Karimi Y.H., Ghesquieres H., Cheah C.Y., Clausen M.R., Cunningham D., Jurczak W., Do Y.R., Gasiorowski R., Lewis D.J. (2024). Epcoritamab in Relapsed/Refractory Large B-Cell Lymphoma: 2-Year Follow-Up from the Pivotal EPCORE NHL-1 Trial. Leukemia.

[3] Karimi Y.H., Cheah C.Y., Clausen M.R., Cunningham D., Farooq U., Feldman T., Ghesquieres H., Lewis D.J., Poon M.L., Lugtenburg P.J. (2026). Efficacy and Safety of Epcoritamab in Relapsed or Refractory Large B-Cell Lymphoma: 3-Year Update from the EPCORE NHL-1 Trial. Ann. Hematol..

[4] Linton K.M., Vitolo U., Jurczak W., Lugtenburg P.J., Gyan E., Sureda A., Leppä S., Chamuleau M.E.D., Gernhardt D., Altıntaş I. (2024). Epcoritamab Monotherapy in Patients with Relapsed or Refractory Follicular Lymphoma (EPCORE NHL-1): A Phase 2 Cohort of a Single-Arm, Multicentre Study. Lancet Haematol..

[5] Salles G., Fox C.P., Hamadani M., Wang A., Gyan E., Sail K., de Castro D.G., Ruan J., Kim T.M., Cheah C.Y. (2025). Indirect Comparison of Epcoritamab Versus Axicabtagene Ciloleucel in Chimeric Antigen Receptor T-Cell-Eligible and -Naïve Patients with Relapsed/Refractory Diffuse Large B-Cell Lymphoma. Clin. Lymphoma Myeloma Leuk..

[6] Engelberts P.J., Hiemstra I.H., de Jong B., Schuurhuis D.H., Meesters J., Hernandez I.B., Valerius T., van der Horst H.J., Gelderloos A.T., Chamuleau M.E.D. (2020). DuoBody-CD3×CD20 Induces Potent T-Cell-Mediated Killing of Malignant B Cells in Preclinical Models and Provides Opportunities for Subcutaneous Dosing. eBioMedicine.

[7] Brooks T.R., Zabor E.C., Bedelu Y.B., Yang X., Karimi Y.H., Nedved A.N., Straubhar A.M., Jang S., Hamilton A.M., Maloy M.A. (2025). Real-World Outcomes of Patients with Aggressive B-Cell Lymphoma Treated with Epcoritamab or Glofitamab. Blood.

[8] Schuster S.J., Huw L.Y., Bolen C.R., Maximov V., Polson A.G., Hatzi K., Armand P., Balakrishnan K., Bartlett N.L., Caimi P.F. (2024). Loss of CD20 Expression as a Mechanism of Resistance to Mosunetuzumab in Relapsed/Refractory B-Cell Lymphomas. Blood.

[9] Duell J., Leipold A.M., Appenzeller S., Fuhr V., Rauert-Wunderlich H., Da Via M., Viardot A., Schmitt A., Stilgenbauer S., Goebeler M.-E. (2024). Sequential Antigen Loss and Branching Evolution in Lymphoma after CD19- and CD20-Targeted T-Cell Redirecting Therapy. Blood.

[10] Lewis C.S., Barraclough A., Hawkes E.A. (2025). Emerging Biomarkers for CD3×CD20 Bispecific Antibodies in Lymphoma. Blood.

[11] Minson A.G., Dickinson M.J. (2025). New Bispecific Antibodies in Diffuse Large B-Cell Lymphoma. Haematologica.

[12] Falchi L., Vardhana S.A., Salles G.A. (2023). Bispecific Antibodies for the Treatment of B-Cell Lymphoma: Promises, Unknowns, and Opportunities. Blood.

[13] Dickinson M.J., Carlo-Stella C., Morschhauser F., Bachy E., Corradini P., Iacoboni G., Khan C., Wróbel T., Offner F., Trněný M. (2022). Glofitamab for Relapsed or Refractory Diffuse Large B-Cell Lymphoma. N. Engl. J. Med..

[14] Budde L.E., Sehn L.H., Matasar M., Schuster S.J., Assouline S., Giri P., Kuruvilla J., Canales M., Dietrich S., Fay K. (2022). Safety and Efficacy of Mosunetuzumab, a Bispecific Antibody, in Patients with Relapsed or Refractory Follicular Lymphoma: A Single-Arm, Multicentre, Phase 2 Study. Lancet Oncol..

[15] Topp M.S., Matasar M., Allan J.N., Ansell S.M., Barnes J.A., Arnason J.E., Michot J.M., Goldschmidt N., O’Brien S.M., Abadi U. (2025). Odronextamab Monotherapy in R/R DLBCL after Progression with CAR T-Cell Therapy: Primary Analysis of the ELM-1 Study. Blood.

[16] Jo T., Hosoya S., Tominaga H., Sakai T., Hayashi S., Matsuzaka K., Shioya H., Noguchi K., Irie S., Taguchi J. (2020). Induction of Effector and Memory Cellular Immunity in a Patient with Long-Term Complete Molecular Response to Philadelphia Chromosome-Positive Acute Lymphoblastic Leukemia. Case Rep. Oncol..

[17] Jo T., Matsuzaka K., Shioya H., Tominaga H., Sakai T., Kaneko Y., Oishi T., Hayashi S., Noguchi K., Irie S. (2020). Mogamulizumab Plus EPOCH Therapy for Patients with Newly Diagnosed Aggressive Adult T-Cell Leukemia/Lymphoma. Anticancer Res..

[18] Jo T., Kubota-Koketsu R., Kaneko Y., Sakai T., Noguchi K., Irie S., Matsuo M., Taguchi J., Abe K., Shigematsu K. (2023). Live Attenuated VZV Vaccination Induces Antitumor Immunity in ATLL Patients. Cancer Immunol. Immunother..

[19] Jo T., Noguchi K., Hayashi S., Irie S., Hayase R., Shioya H., Matsuzaka K., Sakai T., Taguchi J., Abe K. (2018). Long-Lasting Memory of Cellular Immunity in a Chronic Myeloid Leukemia Patient Maintains Molecular Response 5 after Cessation of Dasatinib. Oncol. Lett..

[20] Jo T., Saburi Y., Masunari T., Noguchi K., Sakai T., Taguchi J., Kubota-Koketsu R., Matsuo M., Abe K., Shigematsu K. (2026). Activated Memory Cytotoxic T-Lymphocytes and T-Cell Receptor Vβ Clonality Predict Treatment-Free Remission after Tyrosine Kinase Inhibitor Discontinuation in Chronic-Phase Chronic Myeloid Leukemia: A 1-Year Prospective Immuno-Monitoring Study. Int. J. Mol. Sci..

[21] Alderuccio J.P., Nayak L., Cwynarski K. (2023). How I Treat Secondary CNS Involvement by Aggressive Lymphomas. Blood.

[22] Kline K., Luetkens T., Koka R., Kallen M.E., Chen W., Ahmad H., Omili D., Iraguha T., Gebru E., Fan X. (2024). Treatment of Secondary CNS Lymphoma Using CD19-Targeted Chimeric Antigen Receptor (CAR) T Cells. Cancer Immunol. Immunother..

[23] Mercadal S., Giné E., Karschnia P., Schorb E., Rubinstein P.G., Siddiqi T., Frigault M.J., Soussain C., Nastoupil L.J., Ambady P. (2025). Outcomes of Patients with Primary Central Nervous System Lymphoma Following CD19-Targeted Chimeric Antigen Receptor T-Cell Therapy. Haematologica.

[24] Godfrey J.K., Morgan E.A., Grommes C., Noy A., de Claro R.A., Sabouri-Ghomi M., Bahmanyar E.R., Le K., Wright K., Olszewski A.J. (2024). Glofitamab Stimulates Immune Cell Infiltration of CNS Tumors and Induces Clinical Responses in Secondary CNS Lymphoma. Blood.

[25] Brody J.D., Jørgensen J.M., Belada D., Costello R., Trněný M., Vitolo U., Lewis D.J., Karimi Y.H., Sureda A., Andre M. (2025). Epcoritamab Plus GemOx in Transplant-Ineligible Relapsed/Refractory DLBCL: Results from the EPCORE NHL-2 Trial. Blood.

[26] Falchi L., Sureda A., Leppä S., Vermaat J.S.P., Nijland M., Christensen J.H., de Vos S., Holte H., Merryman R.W., Lugtenburg P.J. (2025). Fixed-Duration Epcoritamab Plus R2 Drives Favorable Outcomes in Relapsed or Refractory Follicular Lymphoma. Blood.

[27] WHO Classification of Tumours Editorial Board (2024). Haematolymphoid Tumours. WHO Classification of Tumours.

[28] Campo E., Jaffe E.S., Cook J.R., Quintanilla-Martinez L., Swerdlow S.H., Anderson K.C., Brousset P., Cerroni L., de Leval L., Dirnhofer S. (2022). The International Consensus Classification of Mature Lymphoid Neoplasms: A report from the Clinical Advisory Committee. Blood.

