# Supplementary material for: Clinical Outcomes and Exploratory Longitudinal CTL/Vβ Repertoire Remodeling in Patients with Relapsed or Refractory Large B-Cell Lymphoma and Follicular Lymphoma Treated with Epcoritamab
Source: Int J Mol Sci. 2026 Jun 5;27(11):5132. doi: 10.3390/ijms27115132 (PMC13257122; doi:10.3390/ijms27115132)
Supplement: Supplementary file 1 [file ijms-27-05132-s001.zip › ijms-4310941-supplementary.pdf]

**Supplemental Table S1. Flow cytometry antibody panel used for CTL subset and TCR Vβ repertoire analyses**

| Assay | Target Population | Marker/specificity | Fluorochrome | Clone/reagent | Manufacturer |
|---|---|---|---|---|---|
| CTL subsets analysis | PBMCs | CD8 | APC | B9.B11 | Beckman Coulter |
| CTL subsets analysis | PBMCs | CD27 | PC7 | 1A4CC27 | Beckman Coulter |
| CTL subsets analysis | PBMCs | CD45RA | ECD | 2H4LD11LDB9 (2H4) | Beckman Coulter |
| TCR Vβ repertoire analysis | $CD8^+$ T-cell | IOTest Beta Mark TCR Vβ Repertoire Kit | – | – | Beckman Coulter |

All antibodies and IOTest Beta Mark TCR Repertoire Kit were purchased from Beckman Coulter (Brea, CA, USA). The kit consists of eight vials containing mixture of conjugated TCR Vβ antibodies specific for Vβ1, Vβ2, Vβ3, Vβ4, Vβ5.1, Vβ5.2, Vβ5.3, Vβ7.1, Vβ7.2, Vβ8, Vβ9, Vβ11, Vβ12, Vβ13.1, Vβ13.2, Vβ13.6, Vβ14, Vβ16, Vβ17, Vβ18, Vβ20, Vβ21.3, Vβ22, Vβ23, covering approximately 70% of the normal human TCR Vβ repertoire.

Abbreviations: APC, allophycocyanin; CD, cluster of differentiation; CTL, cytotoxic T lymphocyte; ECD, phycoerythrin-Texas Red; PBMCs, peripheral blood mononuclear cells; PC7, phycoerythrin-Cy7; TCR, T-cell receptor.
